# Supplementary material for: Decoding RNA Metabolism by RNA-linked CRISPR Screening in Human Cells
Source: bioRxiv. 2024 Jul 26:2024.07.25.605204. Preprint. [Version 1] doi: 10.1101/2024.07.25.605204 (PMC11291135; doi:10.1101/2024.07.25.605204)
Supplement: Supplement 2 [file NIHPP2024.07.25.605204v1-supplement-2.pdf]

bioRxiv preprint doi: https://doi.org/10.1101/2024.07.25.605204; this version posted July 26, 2024. The copyright holder for this preprint (which was not certified by peer review) is the author/funder, who has granted bioRxiv a license to display the preprint in perpetuity. It is made available under aCC-BY 4.0 International license.

Supplementary Figures

Figure S1

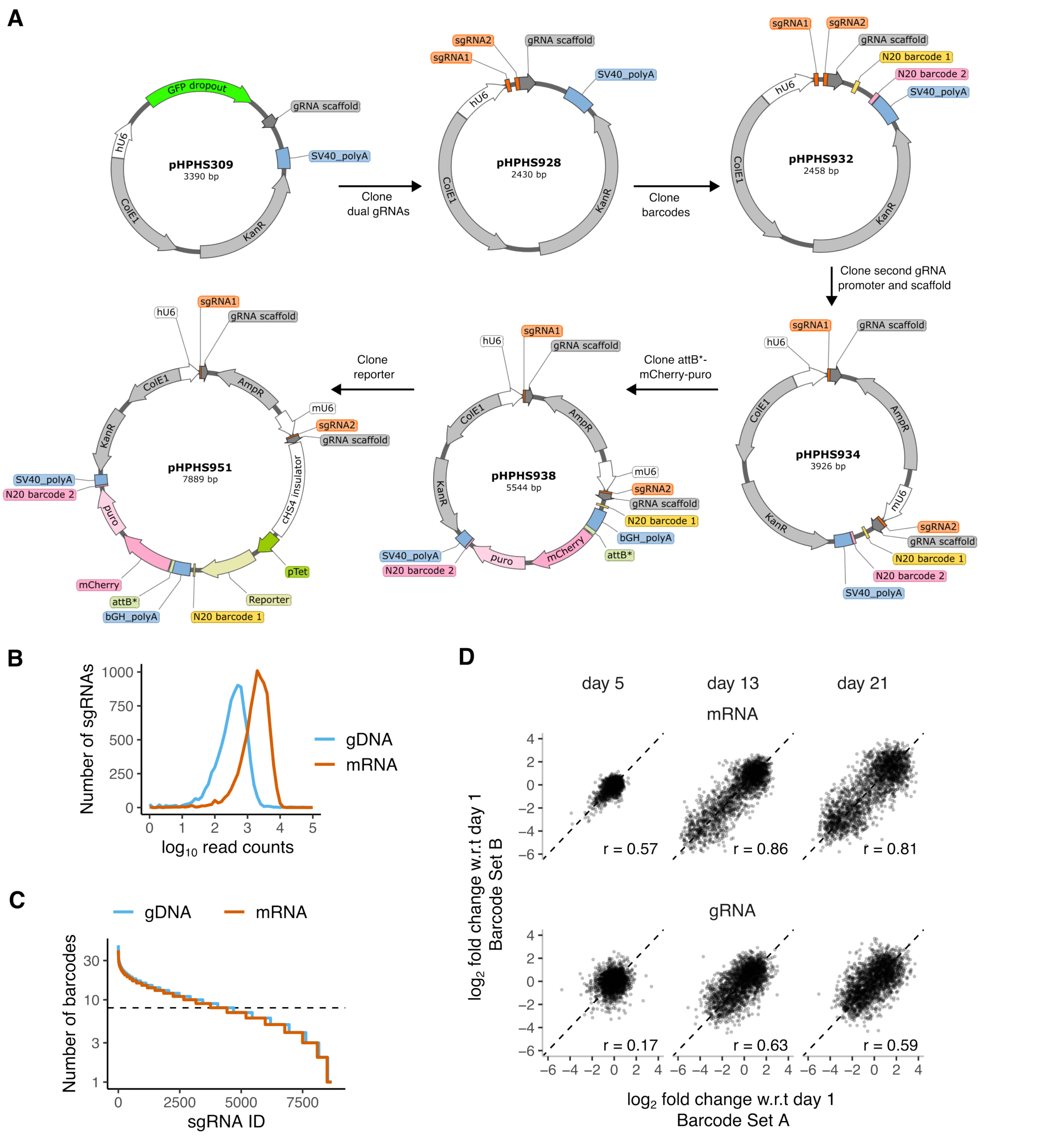

ReLiC library design and validation.

A. *Depiction of cloning scheme for ReLiC library and reporters.*

B. *Distribution of barcode read counts for sgRNA pairs in mRNA and genomic DNA.*

C. *Number of unique barcodes linked to each sgRNA in ReLiC library.*

D. *Correlation between distinct barcode sets in ReLiC fitness screens.* Each point represents a unique sgRNA pair from the ReLiC RBP library. For each sgRNA pair, individual linked barcodes were randomly partitioned into two sets of equal size (or to within a barcode for odd number of detected barcodes). *r* refers to Pearson correlation coefficient between the barcode sets.

bioRxiv preprint doi: https://doi.org/10.1101/2024.07.25.605204; this version posted July 26, 2024. The copyright holder for this preprint (which was not certified by peer review) is the author/funder, who has granted bioRxiv a license to display the preprint in perpetuity. It is made available under aCC-BY 4.0 International license.

Figure S2

A

GO term	Description	FDR	Enrichment
GO:0006364	rRNA processing	1.92E-52	4.08
GO:0006413	translational initiation	1.03E-49	6.16
GO:0022625	cytosolic large ribosomal subunit	3.97E-37	19.6
GO:0022627	cytosolic small ribosomal subunit	3.70E-16	4.72
GO:0000502	proteasome complex	3.44E-05	3.61
GO:0005832	chaperonin-containing T-complex	4.42E-04	9.25

B

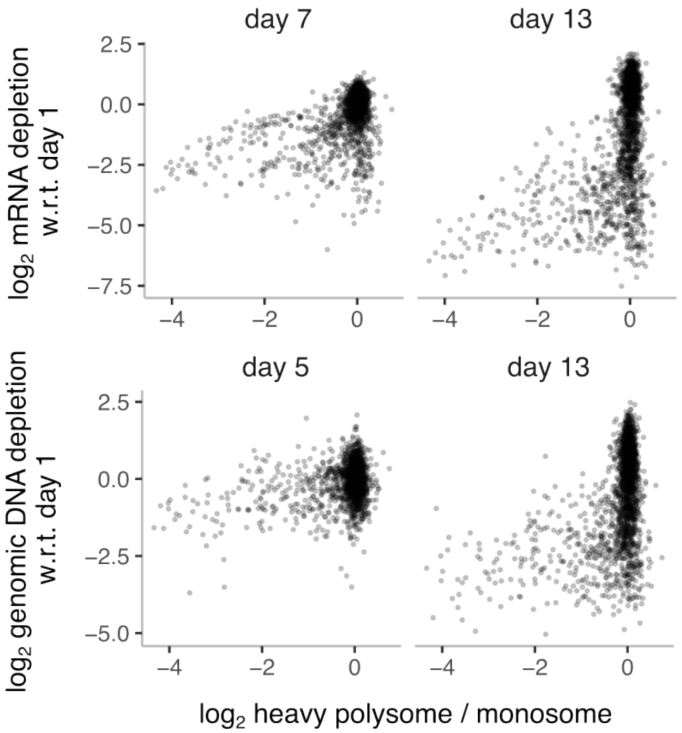

C

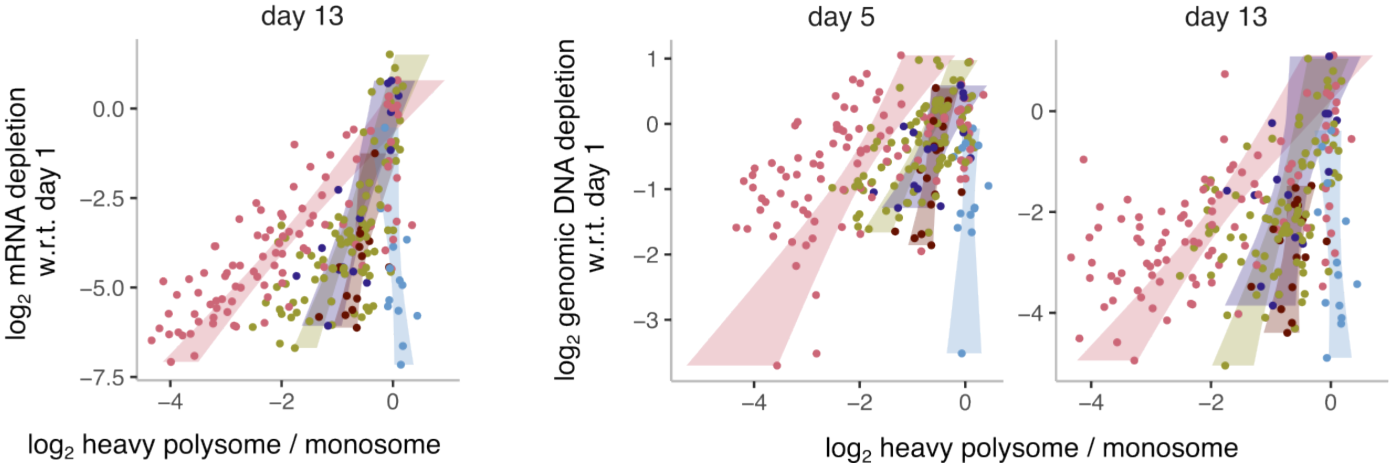

Polysome ReLiC screen for regulators of mRNA translation.

A. *Gene ontology analysis of perturbations that decrease heavy polysome to monosome ratio.*

Gene ontology analysis performed using GOrilla[113] and a subset of enriched terms representative of specific gene classes are shown.

B. *Comparison of heavy polysome to monosome ratio with growth fitness measured by mRNA and genomic DNA barcode seqencing 13 days after Cas9 induction for all gene knockouts.*

C. *Comparison of heavy polysome to monosome ratio with growth fitness measured by genomic DNA barcode sequencing for gene knockouts in specific groups.* Points correspond to genes targeted in the ReLiC-RBP library.

Shaded areas correspond to 95% confidence intervals for a linear fit of polysome to monosome ratio to growth fitness within each gene group.

bioRxiv preprint doi: https://doi.org/10.1101/2024.07.25.605204; this version posted July 26, 2024. The copyright holder for this preprint (which was not certified by peer review) is the author/funder, who has granted bioRxiv a license to display the preprint in perpetuity. It is made available under aCC-BY 4.0 International license.

Figure S3

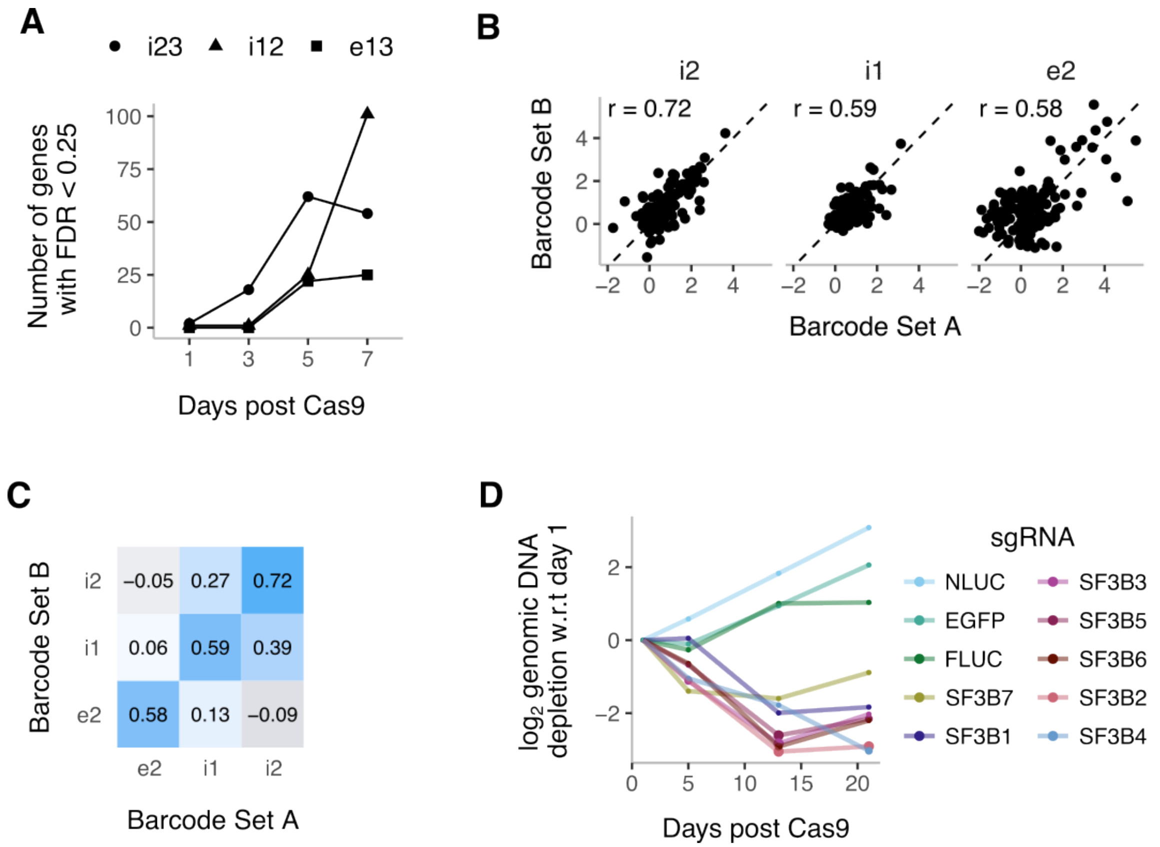

Isoform-specific splicing screen using ReLiC.

A. *Number of gene hits that increase the level of the indicated reporter isoform on indicated days after Cas9 induction.*

B. *Correlation between barcode sets.* For each sgRNA, individual linked barcodes were randomly partitioned into two sets, as in Fig. S1D. Each point represents a unique gene that was classified as a hit either with barcode Set A or barcode set B. *r* refers to Pearson correlation coefficient between barcode sets.

C. *Correlation between relative levels of different mRNA isoforms.* Values represent Pearson correlation coefficients for pairwise comparison between the two barcode sets in B.

D. *Depletion of genomic DNA barcodes corresponding to SF3b complex subunits after Cas9 induction.*

bioRxiv preprint doi: https://doi.org/10.1101/2024.07.25.605204; this version posted July 26, 2024. The copyright holder for this preprint (which was not certified by peer review) is the author/funder, who has granted bioRxiv a license to display the preprint in perpetuity. It is made available under aCC-BY 4.0 International license.

Figure S4

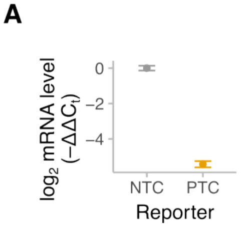

B

GO term	Description	FDR	enrichment
GO:0006413	translational initiation	2.42E-26	7.7
GO:0000184	nuclear-transcribed mRNA catabolic process, nonsense-mediated decay	5.24E-26	4.46
GO:0034198	cellular response to amino acid starvation	1.02E-02	199.33
GO:0036499	PERK-mediated unfolded protein response	1.40E-02	107.33
GO:0042254	ribosome biogenesis	3.19E-02	2.4
GO:0044391	ribosomal subunit	7.64E-14	2.91
GO:0005851	eukaryotic translation initiation factor 2B complex	4.19E-07	155.04
GO:0005850	eukaryotic translation initiation factor 2 complex	9.50E-05	104.65

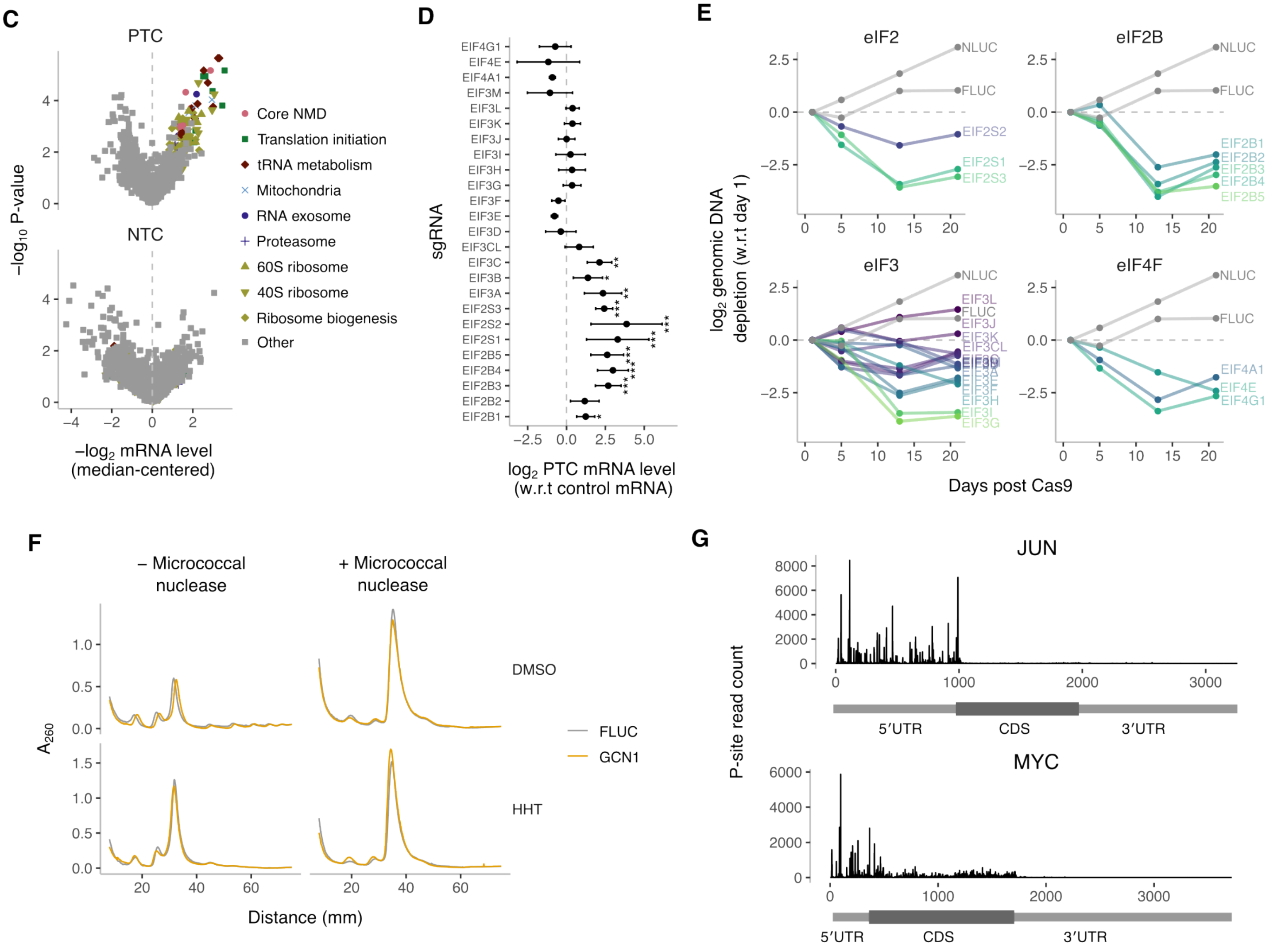

Dissecting mRNA quality control using ReLiC.

A. *Validation of β-globin NMD reporters.* Relative reporter mRNA levels measured by qPCR (n=3). Y-axis represents -$\Delta\Delta C_t$ value of indicated reporter mRNA relative to mCherry-Puro control mRNA.

B. *Gene ontology analysis of perturbations that increase PTC reporter mRNA levels.*

C. *Volcano plot of reporter mRNA levels with dual barcode screen.*

Each point corresponds to a gene targeted by the ReLiC library. Marker shape and color denotes one of highlighted gene groups. Genes with FDR < 0.05 and belonging to one of the highlighted groups are listed in the legend.

D. *PTC reporter levels for individual translation initiation complex subunits.* Points denote mean and error bars denote standard deviation across sgRNAs for each gene. P-values are as calculated by MAGeCK.

E. *Growth fitness after depletion of translation initiation complex subunits.*

(continued on next page)

bioRxiv preprint doi: https://doi.org/10.1101/2024.07.25.605204; this version posted July 26, 2024. The copyright holder for this preprint (which was not certified by peer review) is the author/funder, who has granted bioRxiv a license to display the preprint in perpetuity. It is made available under aCC-BY 4.0 International license.

(continued from previous page)

F. *Polysome profiles of GCN1-depleted and control cell lines after HHT treatment.*
Cells were treated with 1 µM HHT or DMSO for 1 hour prior to lysis. Polysome lysates were digested with 1 U micrococcal nuclease / µg of RNA prior to sucrose gradient sedimentation to isolate RNAse-resistant monosomes and disomes.
G. *Ribosome P-site density on JUN and MYC mRNAs from previous ribosome profiling studies using harringtonine or lactimidomycin to arrest initiating ribosomes.*

bioRxiv preprint doi: https://doi.org/10.1101/2024.07.25.605204; this version posted July 26, 2024. The copyright holder for this preprint (which was not certified by peer review) is the author/funder, who has granted bioRxiv a license to display the preprint in perpetuity. It is made available under aCC-BY 4.0 International license.

Supplementary Table Descriptions

S1: sgRNA pairs and genes targeted in the ReLiC-RBP library

S2: Plasmids used for this study

S3: Oligonucleotides used for this study

S4: Cell lines used for this study

S5: SRA accession numbers

S6: Read counts for sgRNAs

S7: MAGeCK output for sgRNA comparisons

S8: MAGeCK output for gene comparisons
